# Identification and validation of disulfidptosis-related gene signatures and their subtype in diabetic nephropathy

**DOI:** 10.3389/fgene.2023.1287613

**Published:** 2023-11-06

**Authors:** Danping Xu, Chonghao Jiang, Yonggui Xiao, Hanlu Ding

**Affiliations:** ^1^ School of Medicine, University of Electronic Science and Technology of China, Sichuan Provincial People’s Hospital, Chengdu, China; ^2^ Affiliated Hospital of North China University of Science and Technology, Tangshan, China; ^3^ North China University of Science and Technology, Tangshan, China; ^4^ Renal Division and Institute of Nephrology, Sichuan Academy of Medical Science and Sichuan Provincial People’s Hospital, School of Medicine, University of Electronic Science and Technology of China, Chengdu, Sichuan, China

**Keywords:** diabetic nephropathy, disulfidptosis, disulfide stress, machine learning, subtype identification

## Abstract

**Background:** Diabetic nephropathy (DN) is the most common complication of diabetes, and its pathogenesis is complex involving a variety of programmed cell death, inflammatory responses, and autophagy mechanisms. Disulfidptosis is a newly discovered mechanism of cell death. There are little studies about the role of disulfidptosis on DN.

**Methods:** First, we obtained the data required for this study from the GeneCards database, the Nephroseq v5 database, and the GEO database. Through differential analysis, we obtained differential disulfidptosis-related genes. At the same time, through WGCNA analysis, we obtained key module genes in DN patients. The obtained intersecting genes were further screened by Lasso as well as SVM-RFE. By intersecting the results of the two, we ended up with a key gene for diabetic nephropathy. The diagnostic performance and expression of key genes were verified by the GSE30528, GSE30529, GSE96804, and Nephroseq v5 datasets. Using clinical information from the Nephroseq v5 database, we investigated the correlation between the expression of key genes and estimated glomerular filtration rate (eGFR) and serum creatinine content. Next, we constructed a nomogram and analyzed the immune microenvironment of patients with DN. The identification of subtypes facilitates individualized treatment of patients with DN.

**Results:** We obtained 91 differential disulfidptosis-related genes. Through WGCNA analysis, we obtained 39 key module genes in DN patients. Taking the intersection of the two, we preliminarily screened 20 genes characteristic of DN. Through correlation analysis, we found that these 20 genes are positively correlated with each other. Further screening by Lasso and SVM-RFE algorithms and intersecting the results of the two, we identified CXCL6, CD48, C1QB, and COL6A3 as key genes in DN. Clinical correlation analysis found that the expression levels of key genes were closely related to eGFR. Immune cell infiltration is higher in samples from patients with DN than in normal samples.

**Conclusion:** We identified and validated 4 DN key genes from disulfidptosis-related genes that CXCL6, CD48, C1QB, and COL6A3 may be key genes that promote the onset of DN and are closely related to the eGFR and immune cell infiltrated in the kidney tissue.

## 1 Introduction

Diabetes is a chronic disease caused by insufficient insulin secretion or reduced insulin sensitivity. Clinically, diabetes is often classified into four types, of which type 2 diabetes accounts for the largest proportion (Bellary et al., 2021). Due to the lack of treatment methods and its diverse complications, type 2 diabetes has become a huge social burden (National Library of Medicine, 2015). In 2021, the International Diabetes Federation reported that there were more than 500 million people with diabetes worldwide, and this number was expected to exceed 700 million by 2045 (Sun et al., 2022). Unfortunately, diabetic complications are more fatal than diabetes itself (Hu et al., 2023). Indeed, diabetic nephropathy threatens the health of more than 30% of diabetics (Cao and Cooper, 2011; Qi et al., 2017; Yang et al., 2018), often leads to advanced kidney disease (ESKD) and is a common risk factor for cardiovascular disease (Tervaert et al., 2010; Reidy et al., 2014; Tuttle et al., 2014). Globally, diabetic nephropathy is responsible for up to 50% of end-stage renal disease and often leads to disability. (2020; Ruiz-Ortega et al., 2020). Thus, diabetic nephropathy has become a social problem, placing a serious burden on the healthcare system and society (de Boer et al., 2011). Common risk factors for diabetic nephropathy include high blood pressure and obesity, so a good lifestyle and diet may help prevent the disease (Perkins et al., 2019). Over the past few decades, the way people with diabetic nephropathy are treated has changed dramatically (Sawaf et al., 2022), with many patients with refractory disease benefitting from new treatments but a large proportion of patients still progress to advanced disease stages. The pathogenesis of diabetic nephropathy is complex, involving oxidative stress, an inflammatory response, autophagy, programmed cell death and other mechanisms (Duan et al., 2021; Wan et al., 2022; Han et al., 2023; Wei et al., 2023).

Programmed cell death is important for the maintenance of homeostasis and self-renewal (Liu et al., 2022) and is involved in the progression of diabetic nephropathy. Destruction of podocytes in diabetic nephropathy is often accompanied by apoptosis (Bhatt et al., 2016) and macrophages are associated with apoptosis in the kidneys (Jiang et al., 2022). Consequently, baicalein and astragaloside, as well as activation of the PPARγ signalling pathway, can inhibit apoptosis of podocytes in patients with diabetic nephropathy (Lei et al., 2018; Liu et al., 2022). In addition, Metadherin has been shown to accelerate podocyte apoptosis (Liu et al., 2016) and necroptosis has been observed in podocyte damage (Zhang et al., 2021; Zhu et al., 2021), especially in the advanced stage of massive proteinuria (Wang et al., 2022). Wang et al. found that peony glycosides have a certain regulatory effect on necroptosis in podocytes (Wang et al., 2022). Alpha-kinase 1 causes kidney damage through pyroptosis (Cui et al., 2023) and Egr1 may cause podocyte damage by inducing pyroptosis (Kim et al., 2023). Endoplasmic reticulum stress and NLRP3 are also involved in pyroptosis during diabetic nephropathy progression (Wan et al., 2022; Li et al., 2023c). Syringol slows the progression of diabetic nephropathy by inhibiting pyroptosis (Li et al., 2023b). Research on the mechanism of autophagy in diabetic nephropathy is considered extremely valuable (Han et al., 2023). For example, a lack of UCP2 expression will exacerbate podocyte damage (Yang et al., 2023), whereas the tunnelling nanotube/M-sec systems provide protection (Barutta et al., 2023). Ferroptosis is a type of programmed cell death characterised by excessive accumulation of lipid peroxides due to intracellular iron that is significant in the progression of diabetic nephropathy (Wang et al., 2020). The inhibition of ferroptosis can delay the decline of kidney function and improve the prognosis of patients with diabetic nephropathy (Chen et al., 2022b; Feng et al., 2023; Lu et al., 2023; Zhang et al., 2023; Zhao et al., 2023). Similarly, if ferroptosis is activated, it will lead to a deterioration of kidney function (Liu et al., 2023b).

Patients with diabetic nephropathy have a long course of disease and most progress to renal failure, therefore, identifying key genes for early diagnosis of diabetic nephropathy and developing targeted drugs is important. Recently, Xiaoguang Liu et al. discovered a different mechanism of programmed cell death named disulfidptosis (Liu et al., 2023a). This form of cell death is not affected by iron but is closely related to disulphide stress (Zheng et al., 2023). As mentioned earlier, the pathogenesis of diabetic nephropathy is complex and affected by multiple cell death mechanisms, thus disulfidptosis may also be associated with diabetic nephropathy. Therefore, the identification and analysis of key disulfidptosis genes through bioinformatics technology are expected to provide new ideas for the diagnosis and treatment of diabetic nephropathy.

Thus, this study aimed to identify key disulfidptosis genes involved in diabetic nephropathy. The screening of transcriptome data in public databases involved difference analysis, WGCNA analysis, Lasso regression, and SVM-RFE algorithms. The screening results were verified with external data. Immune-related analysis and subtype identification will performed to help select personalised treatment methods for diabetic nephropathy patients. Exploring the correlation between key genes and renal function indicators in diabetic nephropathy further indicates the importance of key genes in disease progression.

## 2 Materials and methods

### 2.1 Data collection and collation

**FIGURE 1:**
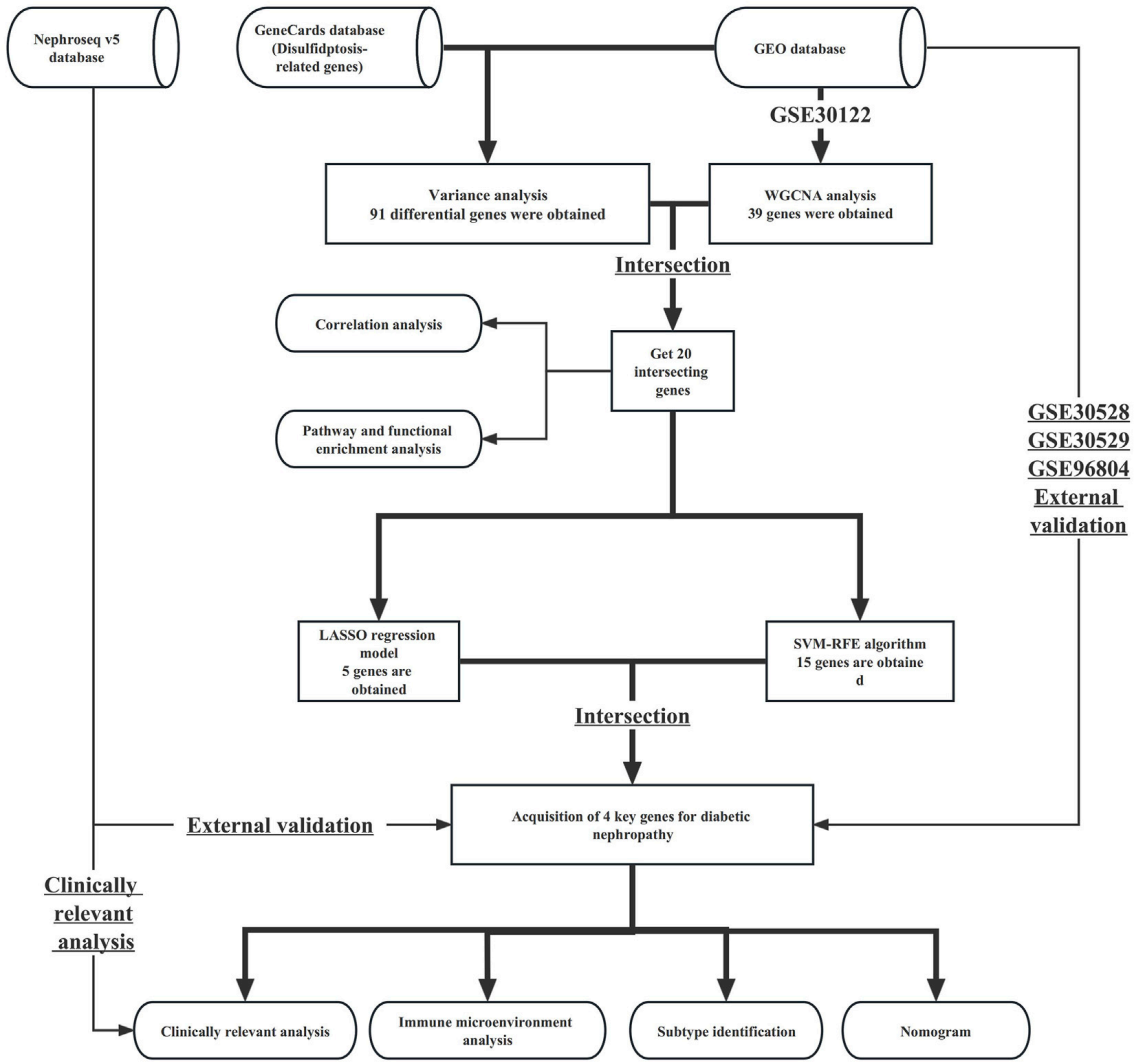
Flowchart of this study.

The study process is shown in the flowchart in Figure 1. The datasets GSE30122, GSE30528, GSE30529, and GSE96804 datasets freely available through the GEO database (https://www.ncbi.nlm.nih.gov/geo/) were used in this study. The GSE30122 dataset belongs to the GPL571 platform, which includes a total of 50 normal samples and 19 samples with diabetic nephropathy. Of the 50 normal samples, 26 were glomerular-sourced, 12 were tubular-derived and the rest were microtubule-sourced, with 9 of the 19 diabetic nephropathy originating from the glomeruli and the rest from the microtubules. The GSE30122 dataset was used as a training cohort, with GSE30528, GSE30529, GSE96804, and other datasets used as external data to verify the diagnostic performance and expression of key genes in diabetic nephropathy. In addition, we retrieved the gene set related to disulphide stress from the GeneCards database and used data from the Nephroseq v5 database to verify the expression patterns of key genes.

### 2.2 Screening and analysis of disulfidptosis-related genes

Perl (Strawberry Edition) was used to annotate the data to obtain a gene expression matrix and the average was used if one gene corresponded to multiple probes. The expression profiles of disulfidptosis-related genes were obtained and screened to identify differentially expressed genes in diabetic nephropathy patients through limma packets. The top 20 differentially expressed genes were visualised through the PheatMap package and co-expression network analysis was performed using the WGCNA package.

### 2.3 Enrichment analysis of functions, pathways, and gene sets

The gene ontology (GO) enrichment analysis was performed to further understand the physiological mechanisms [biological process (BP), cellular component (CC) and molecular function (MF)] of the differentially expressed genes. KEGG enrichment analysis was also conducted to investigate the pathway enrichment using the enrichplot package in clusterProfiler (*p*-value = 0.05, q-value = 1). The enrichment results were visualised using the circlize package in ggplot2. Finally, GSEA enrichment analysis was performed for the disulfidptosis-related genes.

### 2.4 Lasso and the construction of the SVM-RFE model

The minimum contraction operator method (Lasso) and the support vector machine recursive feature cancellation (SVM-RFE) were used to screen the key genes in diabetic nephropathy. LASSO is an algorithm that builds generalised linear models and filters variables. By introducing the penalty term λ, we can build a simpler model. SVM-RFE is a machine-learning algorithm that selects feature variables to gradually delete unimportant trait genes. Finally, the model genes obtained by the two algorithms were intersected as the key genes of diabetic nephropathy.

### 2.5 Construction of nomograms

The basic principle of a nomogram is a multivariate regression model. Each gene was scored according to its contribution to diabetic nephropathy, then the scores were summed to obtain the probability of developing diabetic nephropathy. Nomograms are being increasingly used in clinical practice because they can transform complex regression equations into easy-to-understand graphs. The scale on the nomogram makes it very easy for clinicians to assess the patient’s risk of developing the disease.

### 2.6 External verification of key genes

The LASSO and SVM-RFE results were compared to identify the key genes in patients with diabetic nephropathy. Incorporating key genes into nomograms makes predictions easier to understand and increases the readability of the model. The diagnostic performance of the key genes was verified using three datasets (GSE30528, GSE30529, and GSE96804). The GSE30528 dataset has a total of 22 samples, including 9 diabetic nephropathy samples and 13 normal samples. All samples in the GSE30528 dataset were derived from glomerular cells. The GSE30529 dataset also included 22 samples, including 10 diabetic nephropathy samples and 12 normal samples. All samples in the GSE30529 dataset were derived from tubule cells. The GSE96804 dataset contains 41 glomerular samples from patients with diabetic nephropathy and 20 normal glomerular samples. A ROC curve was plotted for each key gene to infer its diagnostic performance and the differential expression of key genes in the different datasets was analysed. Finally, the expression patterns of key genes were verified in the Nephroseq v5 database.

### 2.7 Comprehensive analysis of the immune microenvironment

Immunotherapy plays an important role in various diseases, so a comprehensive analysis of immune infiltration in patients with diabetic nephropathy was performed to understand their immune characteristics. The infiltration of 28 immune cells in all samples of the GSE30122 dataset was obtained by ssGSEA enrichment analysis. Next, we analysed the differential infiltration of immune cells between groups using R software and visualised the results in a violin diagram. The Cibersort algorithm was then used to estimate the abundance of immune cells. Next, the differences in immune cell abundance between different groups were analysed to determine the relative content of immune cells and the correlation between immune cells, as well as the correlation between genes characteristic of diabetic nephropathy and immune cells.

### 2.8 Identification of molecular subtypes of diabetic nephropathy

The identification of molecular subtypes is particularly important for the personalised treatment of patients with diabetic nephropathy. The ConsensusClusterPlus package was used to type patients with diabetic nephropathy for analysis. Principal component analysis was performed to distinguish patients with different subtypes before the expression patterns of key genes and the immune characteristics of different subtypes were analysed.

### 2.9 Clinical correlation analysis of key genes in diabetic nephropathy

Serum creatinine and glomerular filtration rate are often used as clinical indicators to evaluate renal function, so the correlation between key gene expression and serum creatinine and glomerular filtration rate was analysed using the Nephroseq v5 platform.

### 2.10 Statistical analysis

All statistical analyses were performed in R software (version 4.2.2). A *p*-value<0.05 was considered significant (∗ = 0.05, ∗∗ = 0.01, ∗∗∗ = 0.001).

## 3 Results

### 3.1 Preliminary screening of key genes in diabetic nephropathy

**FIGURE 2:**
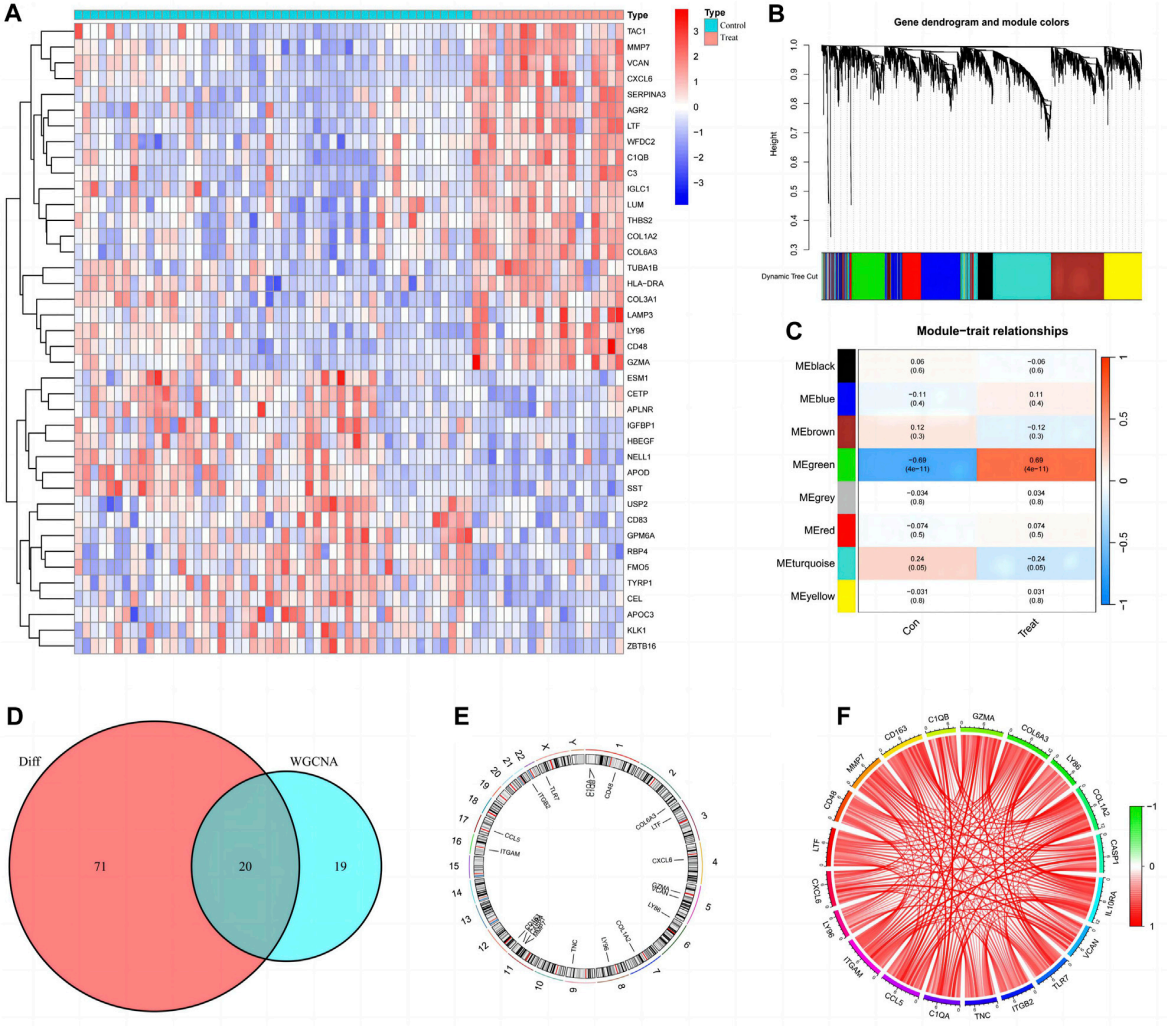
Preliminary screening and analysis of genes associated with Disulfidptosis. **(A)** Differential Disulfidptosis-related genes; **(B)** Differential Disulfidptosis-related genes are clustered into eight modules; **(C)** the MEgreen module shows the most significant difference; **(D)** Intersection of MEgrecn key genes and differential genes; **(E)** Distribution of intersection genes on chromosomes; **(F)** Correlation circle plot of intersecting gene.

Ninety-one differentially expressed disulfidptosis-related genes in diabetic nephropathy patients were identified and the top twenty are presented in heat maps (Figure 2A). The WGCNA analysis identified eight modules relevant for diabetic nephropathy patients (Figure 2B), of which, the turquoise and the green modules were statistically significant (Figure 2C). There were 353 genes in the green module, of which, there are 39 key genes. The intersection of these key genes with the differentially expressed disulfidptosis-associated genes revealed 20 intersecting genes (Figure 2D). The location of the intersecting genes on the chromosomes is shown in Figure 2E. The correlation circle plot of these genes indicated that all genes were positively correlated (Figure 2F).

### 3.2 Enrichment analysis of differentially expressed disulfidptosis-associated genes

**FIGURE 3:**
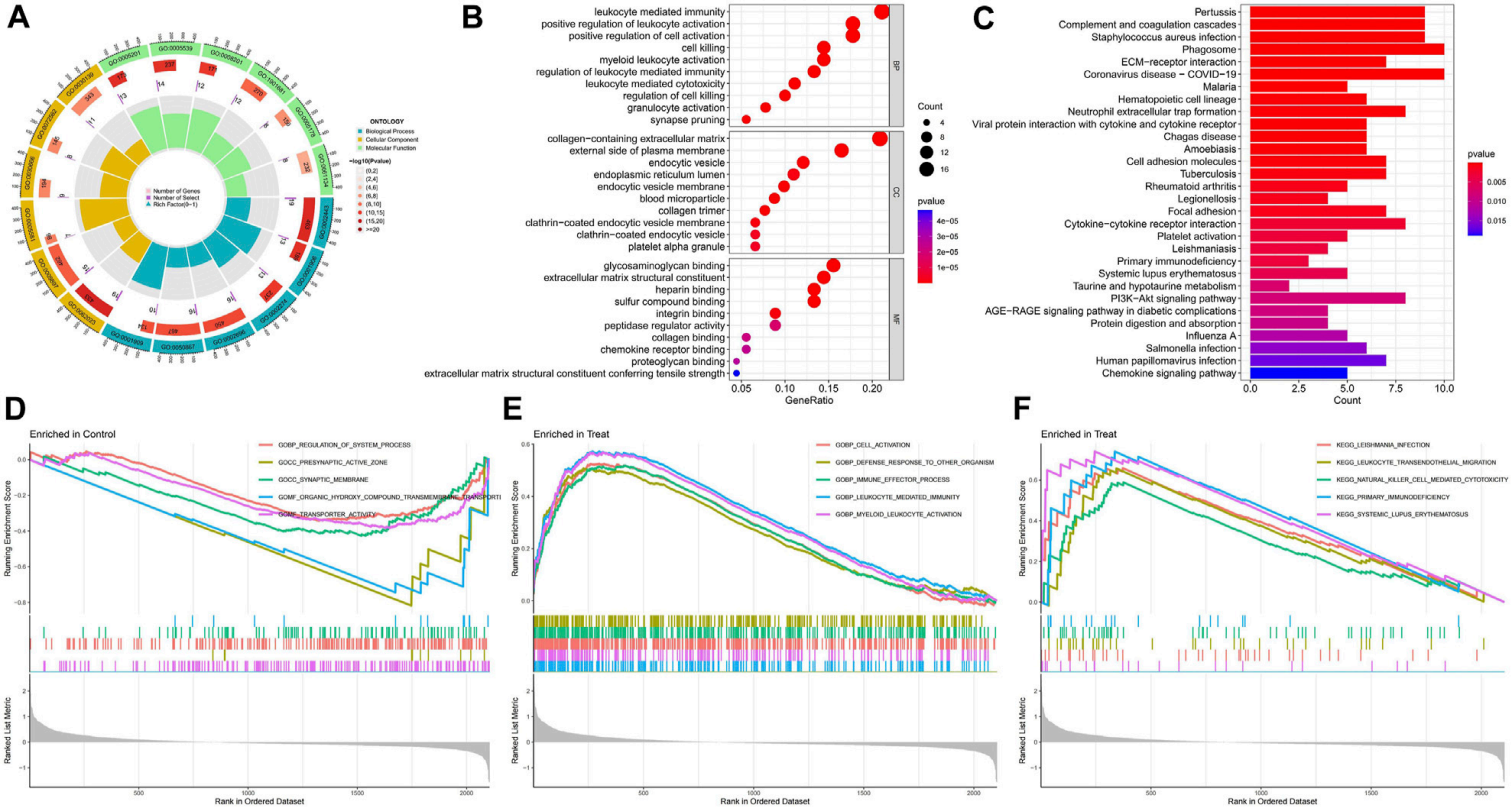
Enrichment analysis results of functions, pathways, and gene sets. **(A)** GO enrichment analysis circle chart; **(B)**GO Enrichment Analysis bubble chart; **(C)** KEGG enrichment analysis bar chart; **(D)** Results of GSEA enrichment analysis of the control group; **(E)** Results of GSEA functional enrichment analysis in diabetic nephropathy group; **(F)** Results of GSEA pathway enrichment analysis in diabetic nephropathy group.

The circle plot of GO analysis provides a visual representation of the enrichment results (Figure 3A), with the outermost part representing the ID and different colours representing different ontologies. The second circle indicates the number of genes enriched, whereas the third circle indicates the number of differential genes enriched. The bubble chart presents the top ten functions of the biological processes (BP), cellular components (CC), and molecular functions (MF) (Figure 3B). The differentially expressed disulfidptosis-related genes are mainly involved in cellular immunity and cell death in various organelles as well as intracellular and intracellular tissue structures and bind to various functional molecules. The KEGG enrichment analysis revealed that these disulfidptosis-related genes were associated with immune activities such as primary immunodeficiency, neutrophil extracellular trap formation, and ECM-receptor interaction. There was also a correlation with COVID−19. The AGE-RAGE signalling pathway is also strongly associated with differential disulfidptosis-related genes (Figure 3C). GSEA enrichment analysis of the normal group showed that it was closely related to synaptic activity (Figure 3D). The GO analysis of the diabetic nephropathy group showed that the differentially expressed disulfidptosis-related genes were strongly associated with the activation of immune cells as well as the defence response (Figure 3E). The KEGG analysis showed that patients with diabetic nephropathy were associated with systemic lupus erythematosus as well as primary immunodeficiency (Figure 3F).

### 3.3 Acquisition of key disulfidptosis-related genes associated with diabetic nephropathy

**FIGURE 4:**
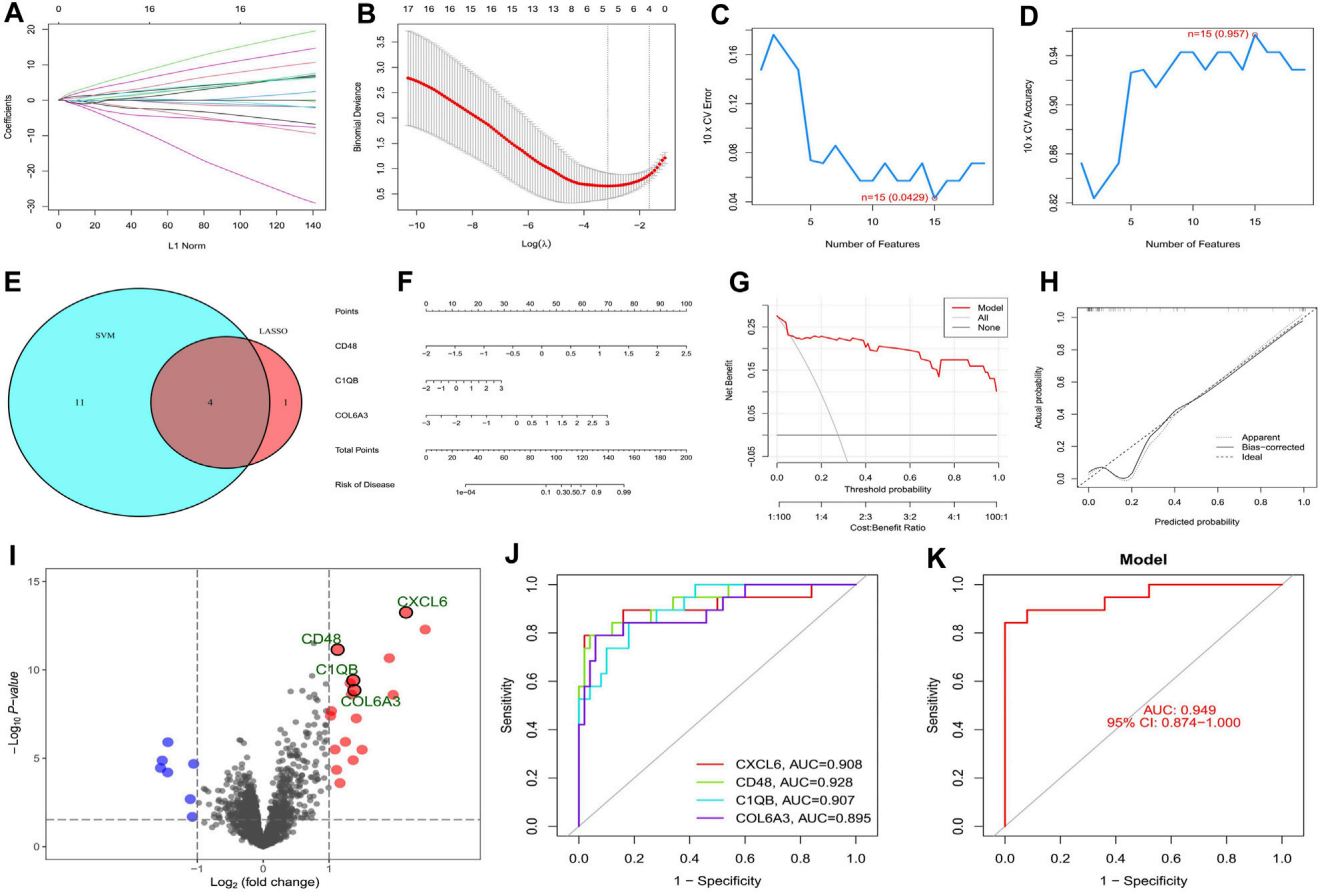
Screening and validation of key genes in diabetic nephropathy. **(A)** Plot of coefficient distribution for LASSO regression; **(B)** Cross-test maps of penalty terms; **(C,D)** SVM-RPF. error and accuracy curve; **(E)** Intersection of LASSO and key genes for SVM-RFE; **(F)** Key genes are involved in constructing the nomogram; **(G)** DCA curve of nomogram; **(H)** Calibration curve for nomogram; **(I)** Differential volcano map of key genes; **(J)** Key gene ROC curves; **(K)** ROC curves of key gene models.

The top twenty intersecting genes were subjected to Lasso and SVM-RFE analysis. In the Lasso regression analysis, the least error was found when 5 genes were taken, and the model was the most concise when 4 genes were taken (Figures 4A, B), so five genes with the smallest error (CXCL6, LTF, CD48, C1QB, and COL6A3) were used for the subsequent analysis. In the SVM-RFE algorithm, 15 genes (CD48, C1QB, CCL5, CXCL6, IL10RA, TNC, VCAN, COL6A3, CD163, ITGB2, C1QA, COL1A2, CASP1, MMP7, and TLR7) gave the smallest error and the highest accuracy (Figures 4C, D). The intersection of the two algorithms obtained four key genes for diabetic nephropathy (CXCL6, CD48, C1QB, and COL6A3) (Figure 4E) which were used to construct the nomogram (Figure 4F). The DCA curve indicates that the nomogram has some application (Figure 4G) and the calibration curve shows the accuracy of the nomogram predictions (Figure 4H). In the volcano plot, CXCL6, CD48, C1QB, and COL6A3 are all highly expressed in diabetic nephropathy patients (Figure 4I), with areas under the ROC curve of 0.908, 0.928, 0.907, and 0.895, respectively (Figure 4J). The area under the ROC curve showed that all four genes had good diagnostic value, with CD48 having the greatest value, so the nomogram with these four key genes shows better diagnostic value (Figure 4K).

### 3.4 Diagnostic performance of key genes in external data

**FIGURE 5:**
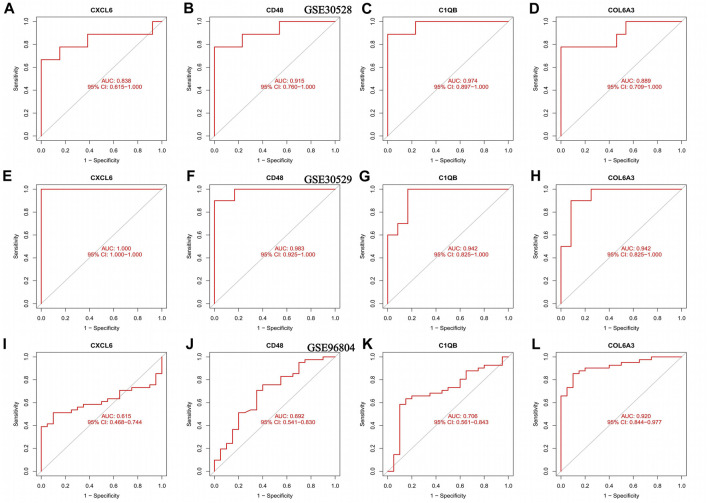
ROC curves for the training set as well as the validation set, **(A–D)** ROC curve of the GSE30528 dataset **(E–H)** ROC curve for the GSE30529 dataset; **(I–L)** ROC curve of the GSE96804 dataset.

The AUC values of CXCL6, CD48, C1QB, and COL6A3 in the GSE30528 dataset were 0.838, 0.915, 0.974, and 0.889, respectively. The 95% confidence intervals were 0.615–1.00, 0.760–1.00, 0.897–1.00, and 0.709–1.00 (Figures 5A–D). The ROC curve areas under CXCL6, CD48, C1QB, and COL6A3 in the GSE30529 dataset were 1.00, 0.983, 0.942, and 0.942, respectively. The 95% confidence intervals were 1.00–1.00, 0.925–1.00, 0.825–1.00, 0.825–1.00 (Figures 5E–H). The ROC curve areas under CXCL6, CD48, C1QB, and COL6A3 in the GSE96804 dataset were 0.610, 0.692, 0.706, and 0.920, respectively. The 95% confidence intervals were 0.468–0.744, 0.541–0.830, 0.561–0.843, 0.844–0.977 (Figure 5I–L). The area under the ROC curve in the external verification data was greater than 0.6, indicating the satisfactory diagnostic performance of the key genes of diabetic nephropathy.

### 3.5 Intergroup validation of key gene expression

**FIGURE 6:**
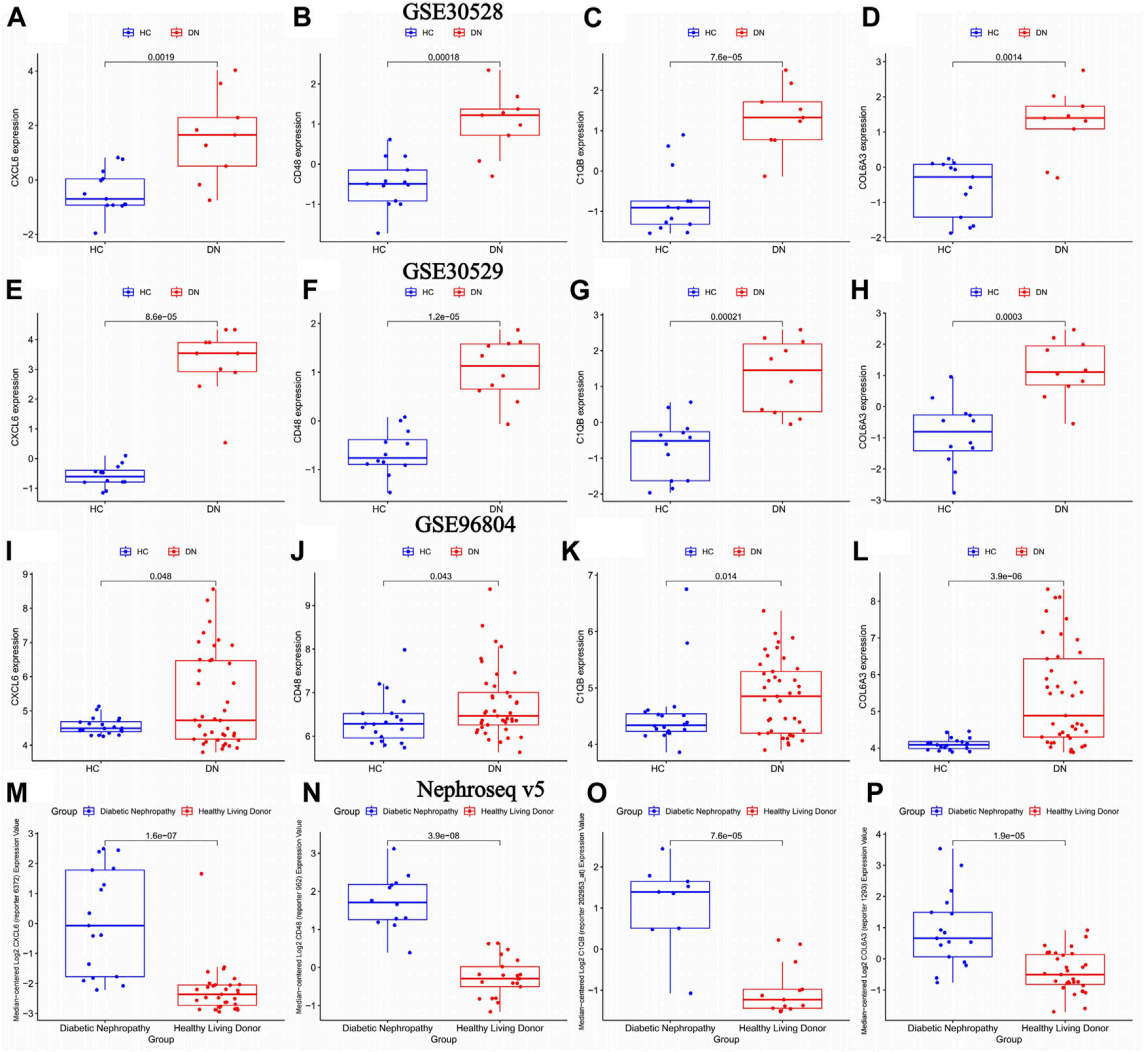
The differential expression of key genes of diabetic nephropathy in the diabetic nephropathy group and the normal group was verified. healthy control (HC): Diabetic nephropathy (DN). **(A–D)** Differential expression of key genes for diabetic nephropathy between diabetic nephropathy group and normal group in GSE30528 dataset; **(E–H)** Differential expression of key genes for diabetic nephropathy between diabetic nephropathy group and normal group in GSE30529 dataset. **(I–L)** Differential expression of key genes for diabetic nephropathy between diabetic nephropathy group and normal group in GSE96804 dataset **(M–P)** Differential expression of key genes for diabetic nephropathy between diabetic nephropathy and normal groups in the Nephroseq v5 database.

The volcano map in Figure 4I showed that CXCL6, CD48, C1QB, and COL6A3 were highly expressed in diabetic nephropathy patients, suggesting that these genes may be strongly related to the onset of diabetic nephropathy. This was verified in three external GEO datasets (Figure 6A–L) and the Nephroseq v5 database (Figure 6M–P).

### 3.6 Immune infiltration analysis in diabetic nephropathy patients

**FIGURE 7:**
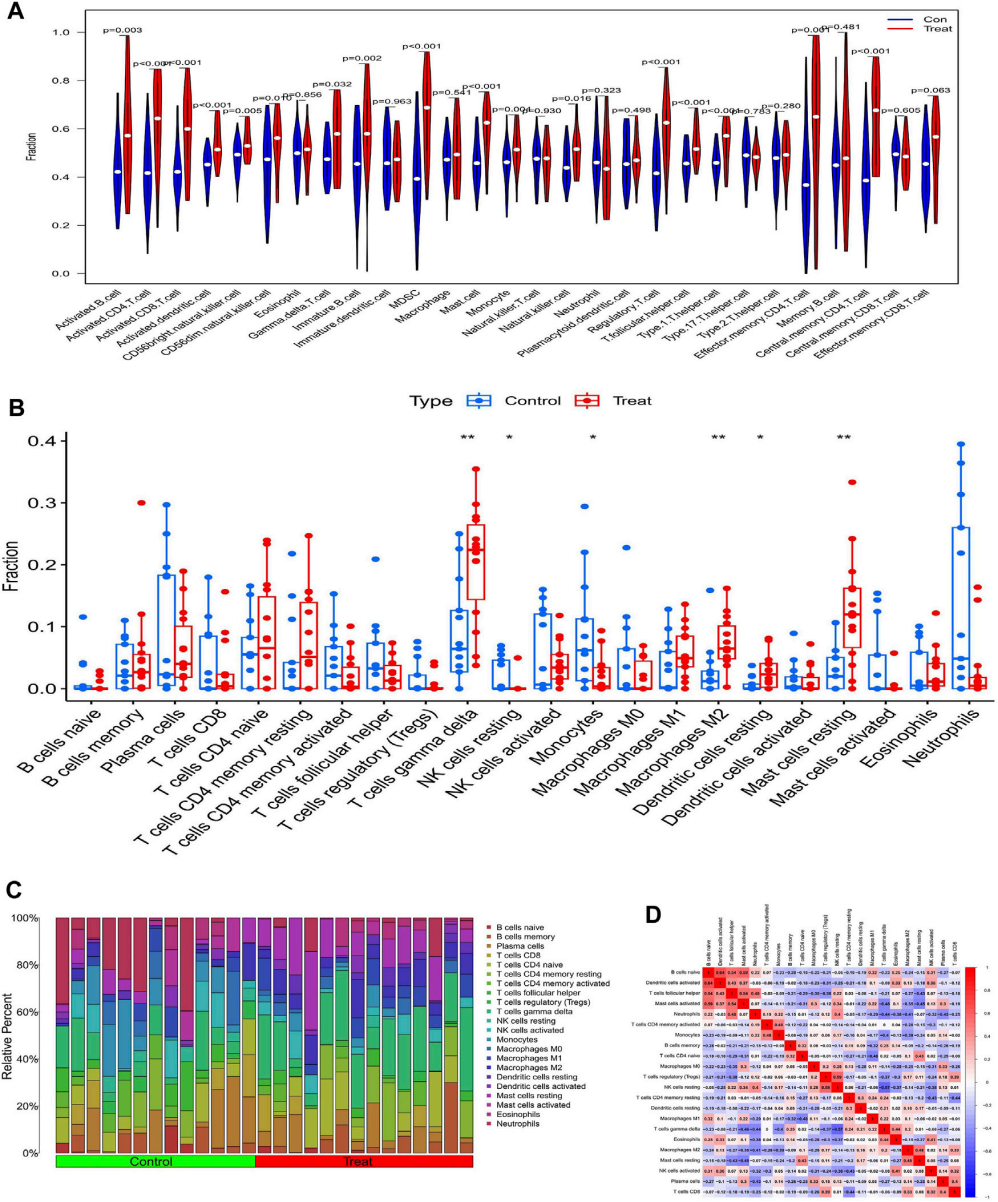
Analysis of immune infiltration in patents with diabetic nephropathy **(A)** Results of ssGSEA immune infiltration analysis in patients with diabetic nephropathy: **(B)** Results of immune infiltration analysis by Cibersort algorithm in patients with diabetic nephropathy, **(C)** Immune cell infiltration ratio bar chart **(D)** Immune cell infiltration-related heat map.

The ssGSEA analysis revealed that almost all immune cells were present in higher amounts in samples from diabetic nephropathy patients than in controls, with the difference in 17 immune cells being statistically significant (Figure 7A). The Cibersort analysis showed that T cells gamma delta, resting NK cells, monocytes, M2 macrophages, resting dendritic cells and resting mast cells were significantly different between the two groups, with only monocytes being more abundant in the normal group (Figures 7B, C). The correlation heatmap shows the correlation between immune cells, where red represents a positive correlation and blue represents a negative correlation (Figure 7D).

### 3.7 Correlation of key genes with immune cell infiltration

**FIGURE 8:**
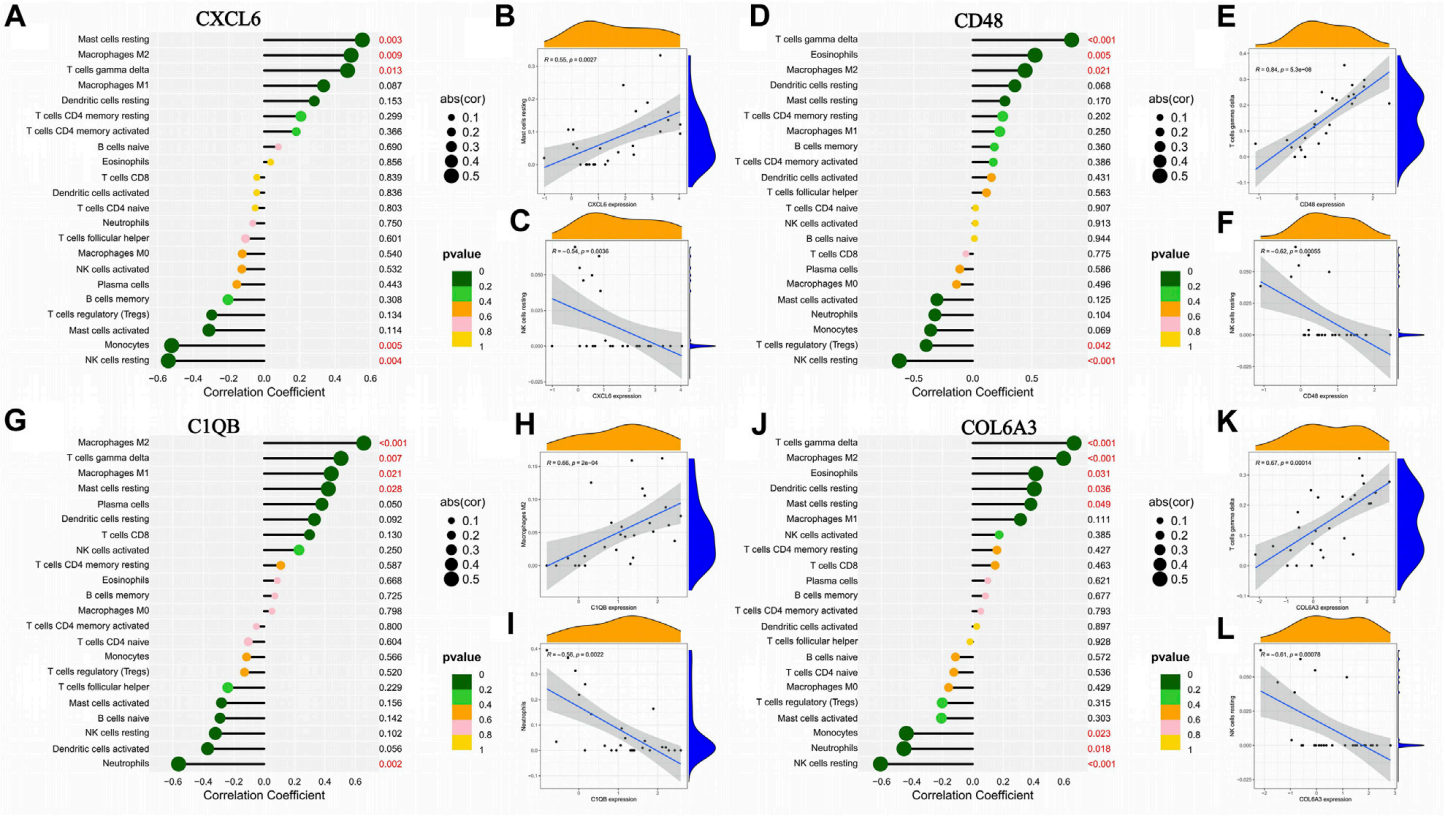
Association between the expression of genes characteristic of diabetic nephropathy and the infiltration of different immune cells. **(A–C)** Correlation of CXCL6 expression with infiltration of different immune cells; **(D–F)** Correlation of CD48 expression with infiltration of different immune cells: **(G–I)** Correlation between C1QB expression and infiltration of different immune cells; **(J–L)** Correlation of COL6A3 expression with infiltration of different immune cells.

The correlation of CXCL6, CD48, C1QB, and COL6A3 with immune cells is shown by lollipop plots, with the most strongly correlated immune cells with key genes shown separately by correlation scatter plots (Figures 8B, C, E, F, H, I, K, L). CXCL6 was positively correlated with resting mast cells, M2 macrophages, and T cells gamma delta and negatively correlated with monocytes and resting NK cells (Figure 8A). CD48 is positively correlated with T cells gamma delta, eosinophils, and M2 macrophages and negatively correlated with regulatory T cells and resting NK cells (Figure 8D). C1QB was positively correlated with M2 macrophages, T cells gamma delta, M1 macrophages and resting mast cells resting, and negatively correlated with neutrophils (Figure 8G). COL6A3 was positively correlated with T cells gamma delta, M2 macrophages, eosinophils, resting dendritic cells and resting mast cells but negatively correlated with monocytes, neutrophils, and resting NK cells (Figure 8J).

### 3.8 Identification and analysis of subtypes

**FIGURE 9:**
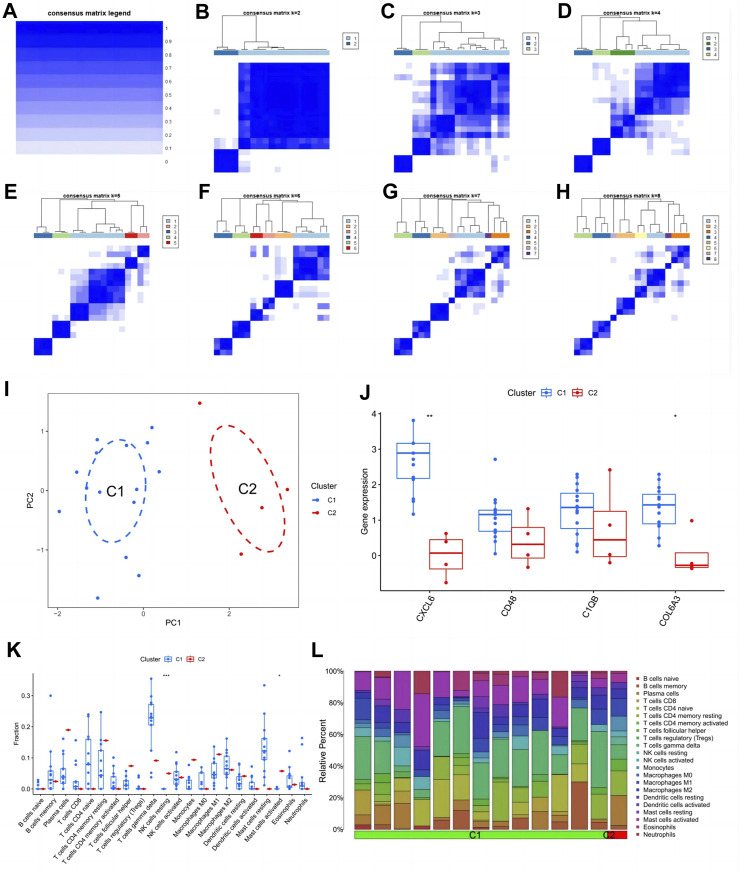
Subtype identification and analysis of diabetic nephropathy. **(A–H)** Matrix heatmap at k = 1,2,3,4,5,6,7,8; **(I)** PCA plot of subtype; **(J)** Expression patterns of key genes in diabetic nephropathy between different subtypes: **(K)** Patterns of infiltration of immune cells between different subtypes; **(L)** The ratio of immune cells between different subtypes.

From the heat map of the classification, we can see that it is most suitable when classifying diabetic nephropathy patients into type 2 (Figures 9A–H). Principal component analysis also showed good differentiation between the two subtypes (Figure 9I). In addition, CXCL6 and COL6A3 expression was significantly different in the C1 and C2 subtypes (Figure 9J), with CXCL6 and COL6A3 highly expressed in the C1 subtype. In the immune infiltration of subtypes, resting NK cells and activated mast cells differed significantly between the two subtypes (Figure 9K), with extensive infiltration of both cells in the C2 subtype. Similarly, the proportion of immune cell infiltration in different subtypes of samples is evident in the scale plot (Figure 9L).

### 3.9 The relationship between key genes and renal function indicators and their expression in various kidney diseases

**FIGURE 10:**
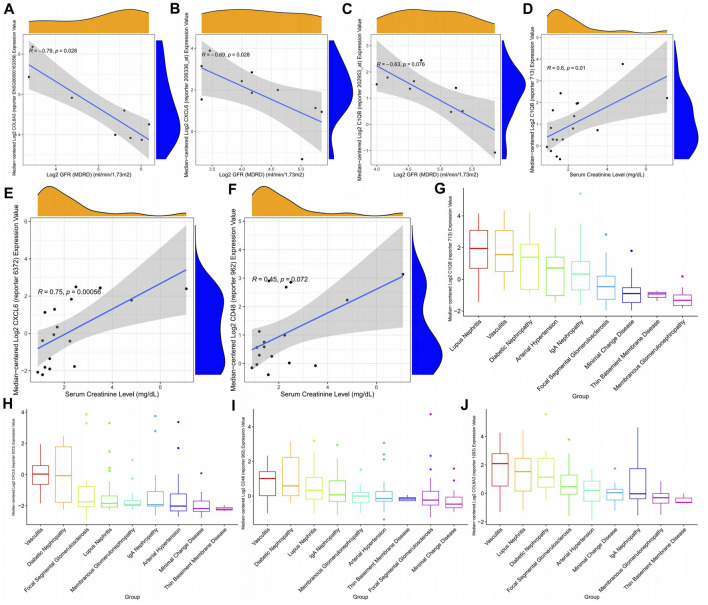
Correlation between diabetic nephropathy-related gene expression patterns and clinical indicators. **(A–C)** Correlation between diabetic nephropathy-associated gene expression patterns and glomerular filtration rate **(D–F)** Association of diabetic nephropathy-associated gene expression with plasma creatinine: **(G–J)** Expression of diabetic nephropathy-associated genes in a variety of kidney diseases.

The glomerular filtration rate of patients gradually decreases as the expression of CXCL6, COL6A3, and C1QB increases (Figures 10A–C), that is, the patient’s kidney function gradually deteriorates with the expression of key genes. In addition, CXCL6, CD48, and C1QB expression were positively correlated with plasma creatinine (Figures 10D–F), indicating the higher CXCL6, CD48, and C1QB expression, the poorer the renal function. Thus, CXCL6, CD48, C1QB, and COL6A3 may be involved in kidney function deterioration in diabetic nephropathy patients. Since there are certain similarities between different kidney diseases, we explored the expression of key genes in different kidney diseases, finding that C1QB was highly expressed in lupus nephritis with the lowest expression in membranous glomerulonephropathy (Figure 10G). CXCL6 is highly expressed in diabetic nephropathy, second only to diabetic nephropathy in vasculitis, and approximately the same expression in other kidney diseases (Figure 10H). CD48 expression is similar to CXCL6 (Figure 10I). COL6A3 maintains high levels of expression in all kidney diseases (Figure 10J).

## 4 Discussion

This study identified four key genes (CXCL6, CD48, C1QB, and COL6A3) involved in diabetic nephropathy using a series of bioinformatics methods. The diagnostic performance of these key genes was confirmed by multiple datasets. The expression of CXCL6, CD48, C1QB, and COL6A3 was higher in diabetic nephropathy patients than in normal controls, indicating that their expression is closely related to disease progression, so these key genes may be potential new therapeutic targets for diabetic nephropathy.

The gradually decreasing glomerular filtration rate and increasing plasma creatinine with the increased expression of CXCL6, CD48, C1QB, and COL6A3 suggests that the deterioration of kidney function in diabetic nephropathy may be closely related to the expression of key genes. Thus, monitoring CXCL6, CD48, C1QB, and COL6A3 expression can help in the diagnosis of diabetic nephropathy. In addition, blocking the expression of these key genes may delay disease progression.

The main function of CXCL6 is to drive inflammatory cells to their destination (Sun et al., 2019a), thus CXCL6 plays an important role in a variety of inflammation-related diseases (Kebschull et al., 2009). Meng-Yao et al. found that CXCL6 can lead to the progression of renal interstitial fibrosis in diabetic nephropathy (Sun et al., 2019b). Interestingly, in a subsequent study, Shen et al. found that ErHuang Formula could delay the process of renal fibrosis in diabetic nephropathy rats by inhibiting CXCL6 (Shen et al., 2019). In addition to causing renal fibrosis in patients, CXCL6 may also be involved in tubular damage (Zeng et al., 2019b; Wang et al., 2021), which explains why the increased CXCL6 expression in our analysis is associated with a deterioration in kidney function in diabetic nephropathy patients.

C1QB is an important protein-coding gene and the protein C1q binds to the proenzymes C1r and C1s to produce C1, the first component of the serum complement system (Reid, 2018). The complement system forms a nonspecific defence mechanism against pathogens in plasma through a proteolytic cascade mediating innate immunity (Daugan et al., 2017). However, overactivating the complement system is associated with impaired kidney function leading to kidney failure (Willows et al., 2020). In addition, it has been proposed that the complement system is involved in the pathogenesis of diabetic nephropathy (Flyvbjerg, 2017). Kelly et al. also found significantly increased C1QB expression in the kidney tissue of diabetic nephropathy patients, and that the classical complement system was activated (Kelly et al., 2015). This is consistent with our results. Fearn et al. reported that the deposition of membrane attack complex formation (MAC) is strongly associated with the progression to advanced stages of diabetic nephropathy (Fearn and Sheerin, 2015). Sun et al. found that patients with more C1q complement deposits had more kidney damage (Sun et al., 2019b). In our study, C1QB was also strongly associated with kidney damage. In addition, many researchers have also found that C1QB may be the pivotal gene in diabetic nephropathy (Xu et al., 2021; Cao et al., 2022; Li et al., 2022; Wang et al., 2023). Peters et al. showed that C1QB can predict a rapid decline in kidney function in patients independently of recognised clinical risk factors for type 2 diabetes (Peters et al., 2017).

The protein expressed by CD48 is a co-stimulatory immunoglobulin receptor on the surface of hematopoietic cells (McArdel et al., 2016). Diabetic nephropathy pathogenesis involves an inflammatory response, cell death, immune response, etc., so CD48 may promote the cascade effect of the inflammatory response through humoral immune regulation, accelerating the progression of diabetic nephropathy (Xu et al., 2021). For example, B cells can lead to the deposition of immune complexes within the kidney through the production of autoantibodies, which can worsen the deterioration of kidney function in diabetic nephropathy (Sosenko et al., 2017). In addition, Li et al. also suggested that CD48 has a high diagnostic value in patients with diabetic nephropathy (Li et al., 2023a).

The main function of COL6A3 is to encode collagen and it is thought to be a profibrotic gene in diabetic nephropathy (Chen et al., 2022a). This may be the mechanism by which COL6A3 causes kidney damage in diabetic nephropathy patients. Zeng et al. also suggested that COL6A3 may be involved in kidney injury in diabetic nephropathy (Zeng et al., 2019a) and Zeng et al. proposed that COL6A3 is a potential diagnostic marker for diabetic nephropathy (Zeng et al., 2019b; Gui et al., 2023).

Validation of key genes across multiple datasets ensures the reliability of our conclusions. However, the research still falls short. Local datasets and experimental validation will make our conclusions more reliable. In addition, further exploration of the expression of key genes in different stages of diabetic nephropathy is expected to provide a more comprehensive perspective for an in-depth understanding of the role of key genes in the development of diabetic nephropathy.

## 5 Conclusion

This bioinformatics analysis identified four disulfidptosis-related genes (CXCL6, CD48, C1QB, and COL6A3) with high diagnostic value and their expression may be closely related to the declined renal function in diabetic nephropathy. Disulfidptosis is expected to become a new therapeutic target for diabetic nephropathy patients, as inhibition of disulfidptosis may delay the deterioration of renal function in patients. The assessment of the immune microenvironment in diabetic nephropathy patients may help personalise their treatment.

## Data Availability

The original contributions presented in the study are included in the article/Supplementary Material, further inquiries can be directed to the corresponding author.
